# Successful switching from risperidone to cariprazine in a schizophrenic patient with pronounced functional deficit. Case report

**DOI:** 10.3389/fpsyt.2023.1155395

**Published:** 2023-03-20

**Authors:** Johan Sahlsten Schölin, José Rodriguez Cruz, Stephan Hjorth

**Affiliations:** ^1^Department of Psychosis, Sahlgrenska University Hospital, Gothenburg, Sweden; ^2^Pharmacilitator AB, Vallda, Sweden

**Keywords:** cariprazine, risperidone, case report, functional deficit, switching, treatment optimization, negative symptoms, clozapine option

## Abstract

A 22-year-old male was admitted to an in-patient psychiatric unit for treatment, after a period of 2 years of increasing psychotic symptoms corresponding to a very severe case of schizophrenia across the entire scale of symptom disorder domains along with some drug abuse comorbidity. Previous treatments with olanzapine (OLA) and risperidone (RIS) had been at best partly successful toward his positive symptoms with no, or even worsening effects on the negative symptomatology. Given the gravity of the latter symptoms and functional impairment of our patient, he might thus have been a candidate for clozapine (CLZ) treatment. It was however decided to switch his antipsychotic treatment to cariprazine (CAR), an agent with a novel pharmacological and clinical profile, because of its favorable pharmacodynamic, pharmacokinetic, and tolerability/safety properties. In a follow-up on the patient 6 months after discharge he is not fully recovered, but the recovery attained reflects a marked functional improvement compared to before the RIS-to-CAR switch. The remarkable response to CAR observed may, speculatively, be in line with the suggestion that CAR could offer an alternative, safer, and more tolerable monotherapy approach (*vs*. CLZ) for patients with severe negative symptoms and functional deficiency resistant to standard antipsychotic treatment. He appears to occasionally still be taking drugs, but no worsening of positive symptoms has been noted. Whether or not he could reach full recovery if he would abstain entirely from drugs of abuse remains an open question.

## 1. Introduction

This case report describes a young patient with severe schizophrenia, previously treated with two different Second-generation antipsychotics with at best limited response. While most antipsychotic agents are capable of attenuating positive symptoms—commonly attributed to dopamine D2 receptor antagonism or partial agonism—the sustained grave functional deficits, associated with self-disorder and disorganized behavior in our patient called for a different pharmacotherapeutic strategy. Accounting for efficacy, safety, and tolerability aspects we therefore decided to try the Third-generation partial dopamine agonist Cariprazine (CAR). This novel agent carries a unique receptor profile, being a partial agonist at dopamine D2 and 5-HT1A receptors but also a high-affinity partial agonist for the D3 receptors. The latter is associated with benefits on negative, cognitive and mood symptoms, including data supporting potential effects on drug use. We detail the clinical findings, rationale as well as the mode of switching, and his status in a follow-up after completing the switch.

## 2. Case presentation

**Figure 1:**
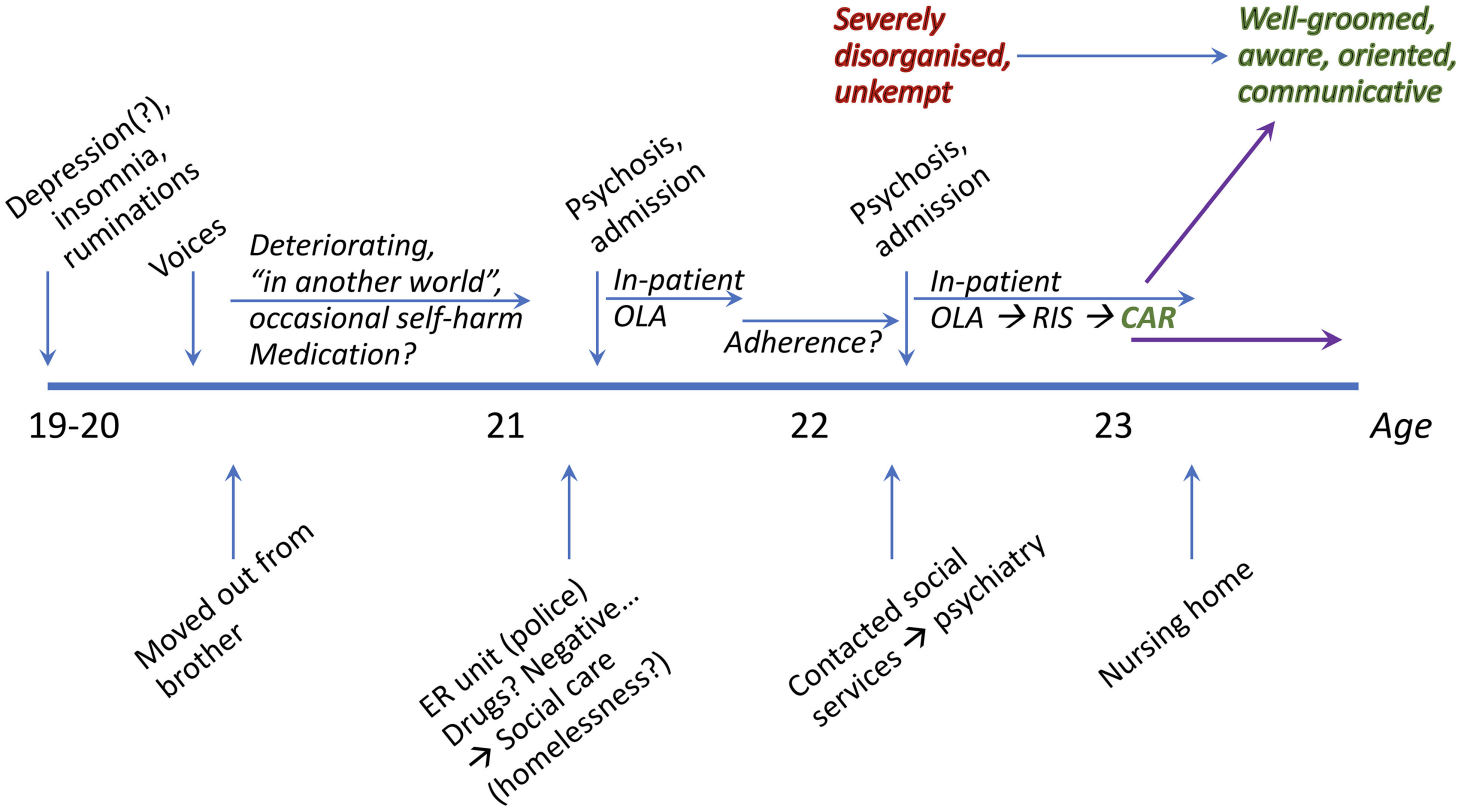
Overview timeline of patient events and medication.

A summary overview of the case presented below is found in Figure 1. A 22-year-old male was admitted to an in-patient psychiatric unit for treatment, after a period of 2 years of increasing psychotic symptoms. He was suffering from auditory hallucinations and disorganized behavior. At the age of 17 he came from the Middle East to Sweden, where he completed his high school studies. Before disease onset there hadn't been any known psychiatric complaints, and there was no known psychiatric illness in the family. In his late teens, he used both alcohol and cannabis in moderate amounts. At age 19 he sought primary care for depressive symptoms. At that time insomnia and ruminations were noted, however not considered to be of psychotic degree. Later that year he developed auditory (voice) hallucinations and described in hindsight his difficulties concentrating. When he was 20, he moved out from his brother's apartment which they previously coinhabited. According to his brother he deteriorated progressively from around the same time, including general loss of function, slowing, anergia and withdrawal. He was described to speak incoherently at times and to appear as “*in another world*”. Occasional self-harm by burning (eventually described to be because of commanding voices).

One year later, at the age of 21, he was taken to the emergency unit by police where he claimed to be using drugs, but screens were negative. He was instead referred to the social-care services, probably due to homelessness. A month later he was forcibly admitted to a closed psychiatric ward (in another region of Sweden than our clinic) due to a psychotic episode. He received olanzapine (OLA; 20 mg), zopiclone (7.5 mg) and propiomazine (25 mg), with general calming and some reduction of positive symptoms achieved by the acute antipsychotic treatment. CT-scan and routine somatic investigations including laboratory tests were unremarkable. After this in-patient care episode, he didn't show for follow-up despite being called multiple times, and his compliance was deemed as poor according to his family. More than half a year later, at the age of 22, he had moved to the city where his brother lived (Gothenburg), which is when he was admitted to our clinic. While contacting social services, they brought him to our unit, where he was judged to be psychotic. According to his brother he didn't use any drugs at this time.

He denied illness, refusing admission and treatment and was admitted involuntarily. At admission he described dysthymia and insomnia, pacing the corridor aimlessly. He couldn't construct adequate sentences. His speech and behavior were severely disorganized to the point of apraxia, and his demeanor disheveled. He came across as disoriented to time and place as well as to the situation, at times even appearing struck by organic confusion. Both formal and emotional contact were impaired. His speech was monotonous, scarce, and lacking in initiative. There was a significant delay in response, short answers, and loose associations, bradykinesia, motor inhibition and objective signs of hallucinations; thus corresponding to a CGI-S score of 7. Somatic status and labs were unremarkable, but a CT scan was not performed because it had been unremarkable <12 months earlier. As plausible determinants both confusion and catatonia were considered; he was however judged to be psychotic, and OLA was switched to risperidone (RIS) with a target dose of 6 mg on day 3. Diazepam and zopiclone were given *PRN*.

The 6-mg dose of RIS given from day 3 resulted in a plasma level of 61 nmol/L, thus within the therapeutic reference range (45–145 nmol/L). Due to insufficient response and symptom severity the dose was anyhow raised to 8 mg on day 14. This only negligibly increased the plasma concentration; to 63 nmol/L on day 18. For the same reasons, another dose adjustment was made to 10 mg on day 28, resulting in a concentration of 114 nmol/L.

After approximately 5 weeks of treatment, he was slightly more communicative and less incoherent. Occasionally he was able to give short answers to questions, but overall, no major status change. At this point, it was evident that previous medication attempts failed to handle the marked degree of positive and negative symptoms as well as cognitive impairments; thus, the drugs used didn't match his pathological profile. Literature and clinical experience suggested that the partial DA agonist cariprazine (CAR) could be a worthwhile choice of drug to target both positive and negative symptom domains, as well as cognitive deficits. With this in mind, we initiated CAR on day 41 (CGI-S: 7) with 1.5 mg, titrating to a target dose of 4.5 mg over 6 days. The tapering of RIS began on day 55 and finished on day 65 (CGI-S: 5; Table 1).

**Table 1:** Medication protocol and CGI scores prior to and upon switching from risperidone to cariprazine.

**Timeline**	**Antipsychotic agent**	**Dose**	**Comments**	**CGI-S**
Day 0			Admission	7
Day 1	Risperidone	4 mg	Start medication	
Day 3	Risperidone	6 mg	Target dose reached	
Day 10	Risperidone	6 mg	*C_*plasma*_* 61 nmol/L (range 45–145)	
Day 14	Risperidone	8 mg	Dose rise (less than expected levels)	
Day 18	Risperidone	8 mg	*C_*plasma*_* 63 nmol/L (range 45–145)	
Day 28	Risperidone	10 mg	Dose rise (less than expected levels)	
Day 37	Risperidone	10 mg	*C_*plasma*_* 114 nmol/L (range 45–145)	
Day 41	Risperidone	10 mg	Overlap switch initiated	7
	Cariprazine	1.5 mg		
Day 47	Risperidone	10 mg	Overlap switch ongoing	
	Cariprazine	4.5 mg		
Day 55	Risperidone	10 mg	Overlap switch ongoing, risperidone down-titration commenced	
	Cariprazine	4.5 mg		
Day 55–65	Risperidone	10 –>0	Risperidone withdrawal phase; cariprazine dose sustained	
	Cariprazine	4.5 mg		
Day 65	Risperidone	0 mg	Overlap switch concluded, risperidone stopped	5
	Cariprazine	4.5 mg		
Day 231	Cariprazine	4.5 mg	6 months after discharge; no positive symptoms	3

On day 56 he was assessed by an occupational therapist. Although obvious impairments in accordance with previous observations persisted, there was also a clear change in status. He initiated conversations spontaneously and showed interest in the other person in a way that hadn't been seen before. His psychomotor inhibition was attenuated, and he displayed a better quality of interpersonal contact.

The patient was discharged 12 weeks after admission. At this point in time, he was well groomed, aware and showed clear consciousness and orientation. He remained psychomotorically inhibited but clearly less so than before. During consultation he mostly looked down but looked up at the interviewer at appropriate times. The thought process and speech remained slow and with low volume, but less so than previously. Hallucinations had diminished to the point of not disturbing during the interview but were still experienced daily. His insight was flawed, his attitude toward medication was ambivalent but he denied side effects.

One month after discharge the patient was staying in a nursing home. He occasionally used cannabis, had no hallucinations and was overall feeling well. Six months after discharge he is still feeling well at the nursing home, with no positive psychotic symptoms noted (CGI-S: 3; Table 1). He works out 1–2 times/week and goes for walks. According to personnel at the nursing home he is more active, including cooking and socializing. He still has a reduced level of function but is satisfied with the medication and reports no side effects.

## 3. Discussion and conclusions

This patient presented with a pronounced functional deficit. His inability to comprehend and organize his exchange with the environment was so prototypically Schizophrenic that it rightfully could be classified as Schizophrenic Autism. This is not to be confused with infantile Autism, but instead a state which on the surface resembles the latter but is in fact a representation of the core pathology of Schizophrenia. It entails a pathological withdrawal from the shared intersubjective world to the benefit of the inner. This inner environment is replete with fears and wishes, fantasies and ruminations. Our patient was at the onset unable to communicate his actual experiences, assumedly due to their intensity and his lack of grip/hold on reality and cognition. To the outside world he was merely devoid of agency and seemingly struck by tormenting positive symptoms. In addition to the observed hallucinatory phenomena that became obvious when interacting with him, his outward appearance and communication clearly implied profound debilitating negative symptoms and a global cognitive impairment. The already full set of symptoms were further accentuated by his disorganization, showing in apraxia for everyday skills such as eating and drinking, even requiring support at the beginning of hospitalization.

Comorbid drug abuse is common in schizophrenia (1). Most drugs of abuse share a capacity to increase mesolimbic dopamine (DA), a neurobiological substrate associated with pleasure and reward processes. Depending on the individual susceptibility (genetic, environmental, stressor, other) and the extent of DA excess triggered, addictive drugs may also lead to overt psychotic states—even in otherwise healthy, non-schizophrenic subjects (2, 3) with presumed intact central DA function. In patients abusing illicit drugs and experiencing psychotic symptoms, the differential diagnosis may therefore be challenging. Although our patient did indeed have a background involving some drug abuse, neither were the quantities consumed nor the consumption coinciding in time to plausibly provide an explanation to his psychosis. Perhaps even more obvious, the clinical presentation of symptoms and signs combined with his poor antipsychotic response collectively questioned the notion of a drug-induced psychotic episode.

At the time of admission in our ward his compliance to the former medication (OLA) was unclear. His illness severity and need for pharmacological treatment optimization was however obvious. Given the previous OLA treatment without clear benefit, and his prominent symptoms in both positive and negative domains we at first decided to replace OLA by RIS. This was motivated by the necessity to alleviate his positive symptoms, under the assumption that they could be dangerous, and without access to all the details due to his lack of communication.

With time it became evident that his negative symptoms were indeed primary in origin, and not secondary due to the positive symptom burden or a misinterpreted depression. We also saw that the response to RIS was unsatisfactory across positive, negative as well as cognitive domains. As he had previously been treated with OLA (albeit of uncertain compliance) without convincing effect, and due to the very poor prognosis of his disease (serious schizophrenia) we wished to choose a drug from a class that had not been tried with this patient before; one with a pharmacodynamically different profile, and potential to access the full range of symptoms. Also, given the patient's young age and an expected long-term treatment (prognosis) the side effect potential of the drug to be chosen needed consideration. These arguments combined lead us to the partial DA D2/D3 receptor agonist CAR as the next drug to be tried.

### 3.1. Pharmacological considerations and rationale

#### 3.1.1. Choice of drug vs. anticipated symptom improvement

Antipsychotic agents share an ability to block the DA D2 receptor, although to a varying degree. The antipsychotic effect of D2 receptor blockade is established and considered pivotal for pharmacological treatment of any psychotic disorder. However, given the wide spectrum of heterogeneity in schizophrenic psychopathology, symptom expressions, and antipsychotic drug responding it is clear that factors beyond D2 blockade impact both the symptom efficacy and side effect profile of agents used in the treatment of psychosis; “one size” does *not* fit all. Often more than one pharmacotherapeutic regimen needs to be tried before the right match is achieved. In addition to efficacy, it is crucial to consider tolerability outcomes.

**Figure 2:**
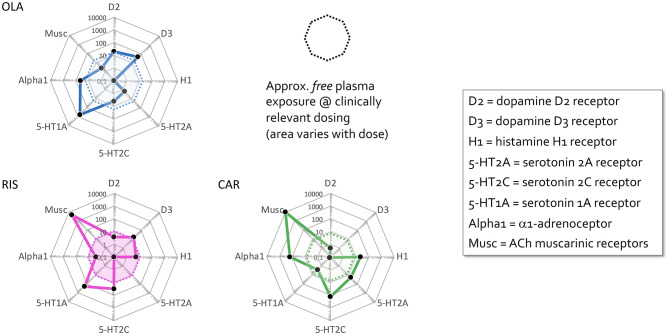
Comparative pharmacodynamic profiles of antipsychotic agents used by the patient. “Cobweb” depiction of pharmacodynamic target profiles of risperidone (RIS), olanzapine (OLA), and cariprazine (CAR) overlaid on the corresponding free (unbound) steady-state plasma concentrations (nmol/L) of these antipsychotics at average clinical dosage (RIS, pink area; OLA, blue area; CAR, green area). Black dots correspond to drug affinities reported in the literature (in nM) for the targets labeled on the edges of the cobweb; the closer to the center, the higher affinity for the target in question. The drug cobweb profiles in this figure were compiled from data in public web databases and complementary literature, including drug SPC's; *viz*. human (cloned or native tissue) receptor affinities (4–6); therapeutic steady-state exposures (7); free fraction of drug plasma concentrations (8). Therapeutic steady-state exposure areas shown were obtained by converting ng/mL (7) to nM, and multiplying by the free fraction in plasma (8) for the corresponding drug [For further detail, see (9)].

The decision to switch our patient from RIS to CAR considered all the above, with the following rationale. Pharmacodynamically, a different receptor action profile was needed. As shown in Figure 2, the pharmacological target profiles of OLA, RIS, and CAR are clearly distinct, therefore aligning with Guideline recommendations to try agents with different target properties when initial antipsychotic pharmacotherapy fails. Specifically, although both OLA and RIS are second generation antipsychotics with strong 5-HT2A antagonist and moderate or strong DA D2 antagonist properties, they display clinically relevant but differential antagonist affinity also for several other targets. In turn, this manifests in diverse efficacy and side effect profiles. For comparison, CAR is a selective and highly potent *partial agonist* for DA D2 and, in particular, D3 receptors—hence unique also among partial DA agonist antipsychotics. Clinically, the compound displays “broad-spectrum” antipsychotic efficacy, addressing positive, negative, cognitive, as well as social functioning symptoms and issues, and with a very benign adverse effect profile (10). Indeed, CAR demonstrated superior efficacy compared to RIS both in treating predominant primary negative symptoms and personal and social performance dysfunction in schizophrenia (11), and has been described as a “*socializing drug*” in this context (12). This profile thus appeared especially attractive for managing the symptoms in our patient, whom on top of severe positive symptoms showed significant negative symptom expressions, including a possible overlap between primary negative symptoms and those secondary to DA blockade (by RIS). It might be argued that clozapine (CLZ) would be a fit for our patient. However, we were concerned that the need for blood monitoring and prominent side effects might result in poor compliance, and therefore decided to try CAR.

Tolerability-wise the side effect potential of the drug to be chosen was also important, particularly given the patient's young age and an expected long-term treatment (prognosis). Compared to older antipsychotics, CAR has little propensity for extrapyramidal side effects, is not sedative, lacks anticholinergic properties, does not produce significant weight gain and metabolic disorder, and does not induce hyperprolactinemia and sexual dysfunction (10, 13). Like other antipsychotic agents, including the partial DA agonist class, CAR may elicit akathisia. This side effect is however typically of mild and transient nature, and very rarely a cause of discontinuation (10).

Yet another factor in the choice of CAR was the comorbid drug abuse in our patient, given the emerging literature on a potential role of agents with DA D3 receptor partial agonist/antagonist properties as a means of treating drug addictive states (14–16). Interestingly, in contrast to another recent case from our practice (17), the introduction of the partial D3/D2 agonist CAR did not appear to change the (sporadic) drug use in this patient. Whether this observation reflects a differential efficacy of CAR against diverse drugs of abuse (e.g., cannabis *vs*. amphetamines) or the extent of abuse(?), remains speculative for the time being.

Lastly, the suitability and choice of formulation and mode of administration need to be weighed in. The choice may be different depending on the phase (acute vs. non-acute) of the illness, and patient preferences. In this regard, there was an obvious pharmacokinetic (PK) benefit of CAR, which together with its active metabolite has a very long half-life [>3 weeks; (18)—even labeled a “*long-acting oral*” antipsychotic (19)]. The long half-life is theoretically advantageous because it limits blood level fluctuations and thereby variations in target profile activity across daily dosing intervals, but also because the occasional missed dose may not be critical to the treatment effect. Since our patient was not positive toward depot injections and compliance is always an issue (even more so with patients with possible substance abuse) CAR thus appeared favorable also from the PK aspect.

#### 3.1.2. Switching process deliberations

When switching from a dopamine receptor D2 blocking agent to a DA receptor partial agonist, certain fundamental principles deserve particular attention. Firstly, pharmacokinetically, switching from a drug with short half-life (e.g., RIS) to a drug with longer half-life (e.g., CAR) there is a risk of a transient drop in receptor occupancy (20). The delay in reaching steady-state with the new drug must thus be considered in order to avoid withdrawal and rebound effects during the switch process. A temporary drop in DA blockade, for example, could mean worsening or relapse of psychotic symptoms. Similarly, the loss of other high-affinity receptor blocking actions of the drug to be withdrawn may result in, e.g., insomnia/agitation (antihistaminergic rebound), sialorrhea/nausea (anticholinergic rebound), or hypertension (alpha1-adrenoceptor blockade rebound).

A second important issue when switching from D2 receptor blockade to partial agonism is the risk of DA supersensitivity phenomena. This is considered a compensatory neuroadaptation resulting from upregulation of said sites and may occur as soon as 3 months on chronic treatment with a D2 receptor antagonist (21, 22). While there is a paucity of clinical information on supersensitivity development following chronic partial DA agonist administration, available preclinical data (23, 24) indicate that this is less likely to occur. Theoretically, the capacity of partial DA agonists to display antagonist or agonist properties depending on the endogenous tone at and state of the receptor will counter the initiation of any sensitivity changes. Supersensitivity may however be more relevant when moving from blockade to partial agonism, as it is yet another potential cause of destabilization during medication change and needs to be handled with care (25), e.g., by temporary benzodiazepine add-on.

Thirdly, a switch involving a change of pharmacodynamic drug profile may have a general unspecific interrupting effect on the frail psychic stability. With all the above in mind, we reasoned that an “overlap and slow withdrawal” switching approach would be the safest option for our patient.

Notably, pre-switch plasma concentrations of RIS were rather modest, despite high clinical dosage. While we have no information as to the drug metabolizing capacity of our patient, 10–29% of individuals with North African/Middle East origin may be CYP2D6 ultrarapid metabolizers (26), possibly underlying unexpectedly low RIS levels relative to doses in our patient.

## 4. Strengths and limitations

The most obvious limitation of any case report is its generalizability to a wider patient population, which must await larger, controlled studies. In addition, whereas a retrospective general rating of illness severity by means of CGI-S was performed, no formal scoring instrument like PANSS, SANS, or similar, was applied to assess the patient symptoms across medications and time. This limits a more rigorous, objective temporal quantification of the treatment effect. However, the detailed description of symptom severity and expression over time, including lack of response to standard antipsychotic regimens which was replaced by substantial, clinically obvious improvement once RIS was switched to CAR provides qualitatively robust evidence for the impact of CAR in this patient. The unknown adherence to the early OLA treatment limits the assessment of its potential treatment effectiveness. It is also possible that social factors and treatment process (e.g., supervised regular use of OLA in a nursing home) could have given a better outcome. However, the documented long-acting favorable pharmacodynamic profile as well as benign tolerability of CAR remained strong arguments to us for selecting this drug before other approaches in this patient. Given the grave negative symptomatology and functional impairment of our patient, he could have been a candidate for CLZ treatment. His remarkable response to CAR may therefore, speculatively, be in line with the suggestion that CAR could offer an alternative, safer, and more tolerable monotherapy approach (*vs*. CLZ) for patients with severe negative symptoms and functional deficiency resilient to standard antipsychotic treatment. Interestingly, while our manuscript was under review, Montgomery et al. (27) published a report on a CLZ-treatment resistant schizophrenic patient successfully converted by switching to CAR monotherapy. They suggested that CAR may present a fruitful alternative treatment in such conditions.

## 5. Conclusions

Our patient represented a very severe case of schizophrenia, with marked symptoms across the entire scale of symptom domains, plus some drug abuse comorbidity. He displayed pronounced psychopathological withdrawal into an inner world, lacking in intersubjective communication with the shared outside. Treatments with OLA and RIS were at best partly successful toward his positive symptoms with no, or even worsening effects on the negative symptomatology. Despite the gravity of the schizophrenic disorder, our data suggest that switching to an antipsychotic agent with a diametrically different pharmacological profile may be worthwhile. To best match the condition of our patient, we chose CAR because of its favorable pharmacodynamic, pharmacokinetic, and tolerability/safety properties. The outcome so far is encouraging. In a follow-up on the patient 6 months after discharge he is markedly, although not fully recovered, but the state attained reflects a significant functional improvement compared to before the RIS-to-CAR switch and we are satisfied with his progress. He appears to still occasionally be taking drugs (when offered by a roommate) but no worsened positive symptoms have been noted. Whether or not he could reach full recovery if he would abstain entirely from drugs of abuse remains an open question.

## Data availability statement

The datasets presented in this article are not readily available because the data are extracted from a patient medical journal and is thus personally confidential within the framework of the medical professionals involved in his treatment. Requests to access the datasets should be directed to JRC, jose.rodriguez_cruz@vgregion.se.

## Ethics statement

Ethical review and approval was not required for the study on human participants in accordance with the local legislation and institutional requirements. The patients/participants provided their written informed consent to participate in this study. Written informed consent was obtained from the individual(s) for the publication of any potentially identifiable images or data included in this article.

## Author contributions

JSS and JRC were in charge of the patient's clinical management and wrote the original draft. JSS, JRC, and SH conceptualized and researched the subject, conceptualized, reviewed, and edited the manuscript. All authors contributed to the article and approved the submitted version.
